# Leveraging Campus Landscapes for Public Health: A Pilot Study to Understand the Psychological Effects of Urban Sheep Grazing on College Campuses

**DOI:** 10.3390/ijerph20021280

**Published:** 2023-01-10

**Authors:** A. Haven Kiers, Kelly M. Nishimura, Carolyn S. Dewa

**Affiliations:** 1 Department of Human Ecology, University of California, Davis, CA 95616, USA; 2 Office of Campus Planning, University of California, Davis, CA 95616, USA; 3 Department of Public Health Sciences, University of California, Davis, CA 95616, USA

**Keywords:** campus landscapes, college student public health, grazing landscape management, mental health in universities, urban grazing

## Abstract

Since the 1980s, college students in the U.S. have self-reported a decline in their physical and emotional health. With these conditions compounded by the COVID-19 pandemic and its physical distancing restrictions, higher education institutions have an increased responsibility to establish strategic interventions and health-promoting programs for their students. Research collaborations between public health professionals and environmental designers have highlighted the benefits of environmental factors, such as wildlife, street trees, and public parks, on mental health. This pilot project aims to build upon the transdisciplinary dialogue between ecology, design, and public health by examining the social benefits of grazing lawnscape management, which is the practice of using herbivorous livestock to manage turfgrass areas. Through the design of an accessible central campus grazing space for a flock of 25 sheep and use of online questionnaires, a smartphone-based single-item survey, and open-ended feedback given via social media, the UC Davis Sheepmower Project addresses three primary questions: (1) Are there differences in self-reported stress levels and well-being between people who did not watch grazing sheep (no sheepmower group) compared with those who did watch grazing sheep (sheepmower group)? (2) Does holding sheep grazing events create opportunities for education about well-being and engagement with the campus community? (3) Can this type of urban grazing installation ultimately contribute to the overall identity of a college campus? Web-based questionnaire results indicate there is no significant difference in self-reported stress levels between the two groups; however, the moment-in-time smartphone-based single item question suggests that the presence of sheep provides temporary, noticeable relief and enhanced mood for those who observe the animals. Reflections posted on social media suggested that participants found the sheep grazing events fostered feelings of community and placemaking within the campus identity. However, the questionnaire sample indicated the grazing events did not have a significant effect on participants’ sense of place or overall campus identity. This transdisciplinary effort breaks down traditionally siloed approaches to human and environmental health and is an example of a whole-systems approach to developing innovative solutions and encouraging applied collective action.

## 1. Introduction

When Frederick Law Olmsted designed Central Park’s Sheep Meadow in the 1850s, he envisioned it as a refuge from the stresses of urban living, where grazing sheep were celebrated as “useful, pastoral features that contributed to the aesthetic and the well-being of New Yorkers” [1]. Combining picturesque aesthetics with inexpensive, practical management, the sheep provided multiple benefits: they created a sense of identity for Central Park, educated and engaged visitors, soothed stressed city dwellers, reduced maintenance labor costs, produced wool to sell, and improved the ecology of the site.

Faced with ongoing health and environmental crises, this Olmstedian model of multifunctionality can serve as a template to redefine campus landscapes as spaces that not only support ecological sustainability and provide opportunities to pilot innovative management operations, but also contribute to the beauty and identity of a site, encourage community engagement, and improve human health and well-being. The Sheepmowers project at UC Davis brings Olmsted’s pastoral sheep back into the spotlight, by utilizing them to maintain landscapes on a college campus.

### 1.1. Grazing Landscape Management

Historically, sheep have been critical in maintaining landscapes, including iconic ones such as those in Central Park and at the White House. Given growing recognition of the need for resource conservation, there is a resurgence of interest in introducing sheep to other sustainable urban landscape systems (for example, to manage vegetation in the narrow aisles of solar farms [2], orchards [3], and vineyards [4]; to improve soil health and reduce the need for tillage in sustainable agriculture [5]; and to target the dry weeds that provide fuel for wildfires in the wildland urban interface [6]. The introduction of sheep into the landscape is a form of grazing lawnscape management (GLM), which is seen as a sustainable alternative to traditional management regimes of gas-powered mowing, fertilizing, and organic waste composting. While sheep produce methane (CH4) and nitrous oxide (N20), Lenaghan (2016) [7] analyzed the greenhouse gas emissions of both conventional and grazing landscape management systems and found that a grazing regime can reduce net emissions by 34–37% (980 kgCO2e/ha/year). In addition to providing an environmental (and cost-effective) alternative to conventional maintenance regimes, contributions of sheep to the urban landscape may also include cultural and social benefits, such as promoting student mental health and well-being and affording opportunities for education and engagement.

### 1.2. Rising Mental Concerns among College Students

The college experience is meant to be a journey of self-discovery and personal growth, a passage to adulthood. However, challenges related to the new experiences, living situations, and academic and financial pressures of college increase student vulnerability to mental health challenges, including stress, anxiety, depression, and social and emotional loneliness [8,9]. Issues of poor mental health among college students have increasingly become a critical public health concern [10,11,12,13,14], with U.S. college students’ self-reported physical and emotional health steadily declining since the 1980s [15]. Conditions worsened during the COVID-19 crisis, when a combination of fear, repeated quarantines, and social isolation as a result of the pandemic took a severe toll on the psychological well-being of individuals [16]. The pandemic and its accompanying effects profoundly impacted collegiate mental health [17,18,19]. One US study, the Healthy Minds Survey 2020, found that nearly a fifth of the sample of 33,000 college students from across the country reported moderately severe or severe depression, and nearly two-thirds of students were struggling with loneliness and feeling isolated as a result of the COVID-19 pandemic [20,21]. Globally, college students were forced to participate in classes almost exclusively online, resulting in higher rates of academic stress [22]. Restrictions requiring students to self-isolate at home or in dorm rooms resulted in increased rates of loneliness and a lack of perceived social support, both of which are harmful to mental health [23].

Colleges and universities play an essential role in providing support to college students and serving their health, education, and safety needs. Even before the onset of the pandemic, universities were increasingly being asked to address the growing problem of stress and anxiety in their student populations. As mental health issues continue to rise on campuses, college health clinics and counseling services have been struggling to keep up with the demand [24]. Outreach programming is one method that allows counseling centers to reach out to university communities with programs to promote mental health that help students to cope with emotional challenges while decreasing the likelihood that they escalate into crises by serving in an educative and preventive capacity [25]. Mental health outreach collaborations with professors, staff, students, and other campus organizations can reach students by reducing the stigma of seeking help [26].

### 1.3. Campus Nature Rx (CNRx) to Promote Mental Health

The Campus Nature Rx (CNRx) approach is an example of an outreach programming partnership that is gaining traction at universities. Based on the belief that the university experience relies on more than academia, CNRx programs address student mental health through activities that promote a connection to the natural world and support a sense of place and belonging [27]. Such programs are supported by a growing body of research demonstrating reduced stress levels and decreased feelings of depression after spending time in nature (e.g., Ulrich 1979; Bratman et al., 2015; Ward Thompson et al., 2016, Frumkin 2017) [28,29,30,31]. Healthcare providers have increasingly begun promoting the healing power of time spent in nature through the concept of Park Prescriptions, the practice of healthcare providers “prescribing” parks and outdoor spaces to patients [32], leading to the creation of outreach programs such as Park Rx, Nature Rx, Nature as Medicine, and others.

Studies have also looked into how spending time in nature specifically affects college students. For example, research shows that spending between 10 and 20 min in nature can improve mood by an average of 86% [33], and a 1–5 min “microbreak” in campus greenspaces can reduce levels of stress [34]. Kiers et al. (2021) [35] found that students attributed their participation in a Nature Rx course with reduced stress levels and strengthened social connections. Such classes and programs promote well-being through a combination of mental health outreach, opportunities for student engagement, collaborations with university staff and faculty, and, perhaps most importantly, contact with campus greenspaces. Additionally, research has shown that people who spent time outdoors during the pandemic reported better psychological health outcomes than those who did not go outside [36,37].

### 1.4. Animal-Assisted Interventions

Higher education campuses have also begun exploring animal-assisted interventions as an approach to support their students. “Animal-assisted intervention” is a broad term that describes the utilization of animals in a patient’s treatment process as a means to introduce positive effects on human health and well-being. These therapeutic interventions can come in many forms. This includes interacting with certified therapy dogs and their handler on a “drop-in basis” [38,39]; interacting with guide dogs in training during 10–15 min sessions [40]; and caring for and managing farm animals [41,42]. There is a growing body of research that suggests the positive emotional response of animal-assisted therapy on participants, including significant decreases in self-reported anxiety and loneliness [38,42], reduction in depression symptoms [42,43], and improved self-esteem and coping abilities [42]. Many of these studies suggest that lower levels of anxiety may have been the result of the unstructured and voluntary nature of their respective outreach programs, allowing participants to interact with animals at a comfortable pace and gain counseling support without committing to formal services. Likewise, these informal outreach programs likely helped facilitate a non-threatening environment that allowed participants to socialize and reduce a sense of loneliness [38].

The UC Davis Sheepmowers are one of the foundational elements of a broader, campus-wide Nature Rx effort to create programs and landscapes that support a more holistic vision of sustainability that encompasses human health and well-being at its core. The project was founded on the belief that while people are engaged in immersive outdoor activities, they are also reaping the health and well-being benefits of being in nature and experiencing the value of people-animal interactions. This pilot study examines the questions: (1) Are there differences in self-reported stress levels and well-being between people who did not watch grazing sheep (no sheepmower group) compared to those who did watch grazing sheep (sheepmower group)? (2) Does holding sheep grazing events create opportunities for education about well-being and engagement with the campus community? (3) Can this type of urban grazing installation ultimately contribute to the overall identity of a college campus?

## 2. Materials and Methods

### 2.1. Study Design

The UC Davis Sheepmowers are a flock of 25 university sheep that graze in a fenced 1-acre lawn area on the central UC Davis campus. First launched during the COVID pandemic in May 2021 (when the majority of instruction was still remote), an initial goal of the project was to test the effectiveness of sheep in maintaining campus lawns. However, after noting the number of students, staff, and faculty attending the sheepmowing events and the positive reactions (e.g., increased Instagram followers and trending hashtags) the sheep elicited, researchers expanded the pilot study the following year to begin to understand the effects of sheep grazing on-campus mental health and well-being in anticipation of future studies.

Grazing events for this pilot mental health evaluation began in the spring of 2022 and occurred approximately once every three weeks for three days at a time on Wednesdays, Thursdays, and Fridays from March through June (3/30–4/1, 4/25–4/27, 5/18–5/20, 6/8–6/10). Following the Approved Protocol for Animal Use and Care (#21902), approximately 25 mature ewes, a mixture of Suffolks, Hampshires, Dorsets, and Southdowns from the UC Davis Sheep Facility (located less than a mile—or approximately 1.5 km—away), were loaded onto a trailer and transported to the grazing site on central campus for each grazing event. The sheep typically grazed between 9 a.m. and 3 p.m. and had continuous and unlimited access to a source onsite that provided fresh drinking water. While at the grazing site, the sheep were under constant supervision by at least two student shepherds. At the end of each day, the sheep were loaded onto the sheep facility trailer and hauled back to the Sheep Facility for the night.

**Figure 1:**
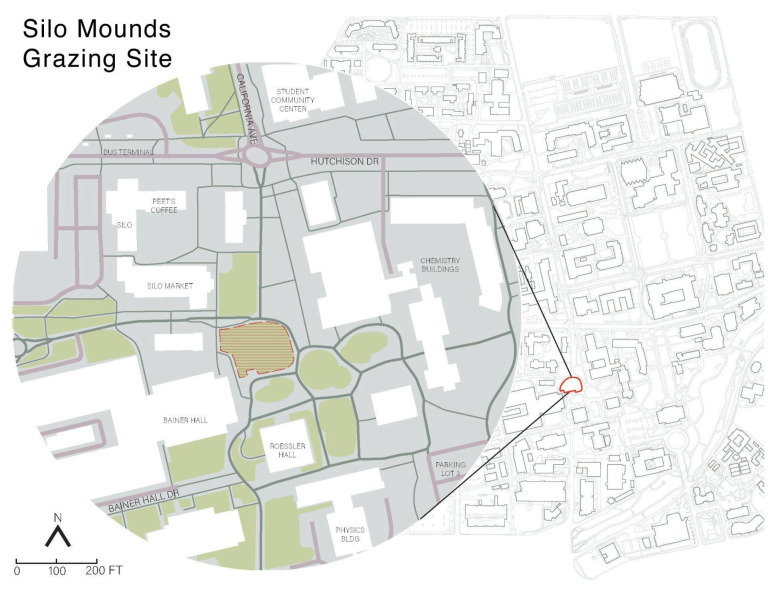
UC Davis campus map, with grazing site enlarged.

**Figure 2:**
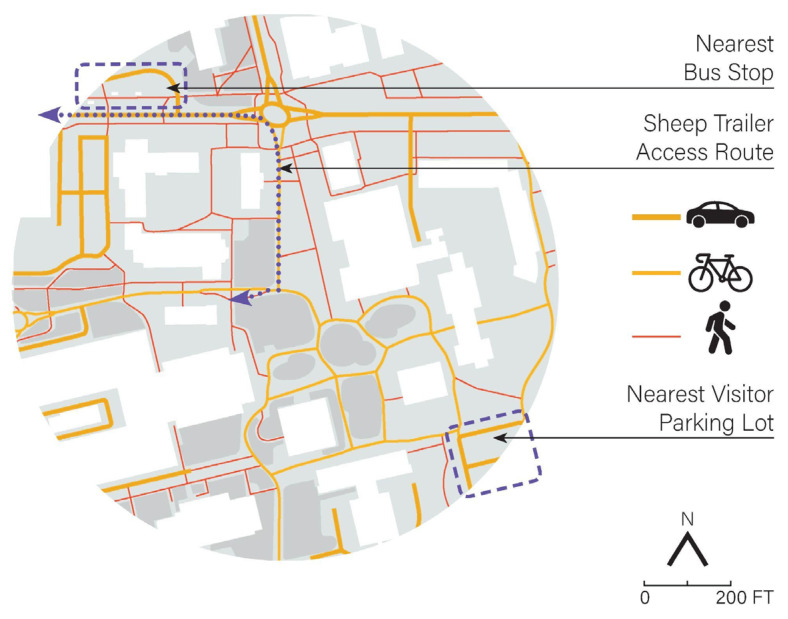
Site accessibility.

The project site, known as the “Silo Mounds” (Figure 1), is a centrally located lawn in a high-traffic area at the campus core surrounded by the engineering and chemistry buildings. Designed by modernist landscape architect Lawrence Halprin in 1967, the amoeba-shaped space rises approximately 4′ (1.2 m) to form a wide grassy mound ringed by pedestrian walkways and a bike path. Adjacent vehicular roads require gate code access, and the nearest parking lot is several minutes away by foot. Because accessibility to the site from outside of the university is somewhat limited (Figure 2), the vast majority of visitors to the site were affiliated with UC Davis, either as students, staff, or faculty. Visits to the sheep grazing events were designed to be informal and unstructured. Any participation or socialization was voluntary and at a self-guided pace. Likewise, the outdoor event favored participants to physically distance themselves and practice COVID-19 preventative measures.

The site was secured with an electric perimeter fence run by solar power and clearly marked with warning signs for the public. Wooden perimeter snow fencing provided a secondary boundary in case sheep escaped from electric fencing. The snow fencing, which was set up approximately 5′ (1.52 m) away from the electric fence, also protected pets and people from getting too close to the electric fence and provided a buffer between the sheep and their visitors. The snow fence was left onsite for the duration of the spring quarter; the electric fence was installed for each event. Student shepherds, trained in animal safety and handling procedures, were in charge of the set up and cleaning of the site. In addition to ensuring the health and well-being of the sheep, student shepherds were charged with herding the sheep back into their trailer at the end of each grazing day.

Examining the temperature and conditions at 12:53 p.m. on the days on which data were collected suggests that weather conditions were similar on days when the sheepmowers were and were not present (Table 1).

### 2.2. Survey Methods

A pragmatic evaluation design was employed to understand the effects of the sheepmowers on observers [44,45,46]. As such, experimental controls in terms of sample selection, environment, and length of exposure to the sheepmowers were not introduced. Rather, this study examined the perceived effects of the sheepmowers under “real world” conditions that would exist if they were introduced into a university setting. Two groups of participants were asked to complete questionnaires. One group consisted of those who came to observe the sheepmowers. Responses from this group were compared to those from a second group comprised of those who were passing through the sheepmower area on a day on which the sheepmowers were not present.

Data were collected using two types of questionnaires in this study. One was a 31-item web-based questionnaire. The second was a smartphone-based single-item question. Open-ended questions via social media were also used to collect reflections on experiences watching the sheepmowers.

#### 2.2.1. Web-Based Data Collection

The online survey was open for the spring grazing season from April to June 2022, during which time four sheep grazing events took place. The survey was developed by members of the research team. The team developed two surveys on the Qualtrics platform, one to be completed by those who attended the sheepmowing event, and a second one for the comparison group (potentially made up of different people) who completed it during the same time period at the same site on days when the sheep were not grazing. The first survey (IRB Protocol #1894378-1: Exempt) consisted of 16 questions covering the following topics: (1) Overall life satisfaction, (2) Current well-being, (3) Place attachment and belonging, (4) Use of outdoor space, (5) Experience at the Sheepmower event, and (6) Demographics. The comparison group survey asked the same questions as the other, with the omission of Section 5, “Experience at the Sheepmowers Event”.

Researchers utilized QR codes on clipboards and posted signs at the grazing site to direct visitors to the online survey when the sheep were present. There were 203 survey responses on days when the sheep were grazing. Posted signs with QR codes placed at the site when the sheep were not present directed survey participants to the online control survey. The signs included a photo of a toy stuffed sheep that people could enter to win if they took the survey. Upon completion of the survey, participants were directed to a separate website where they were invited to enter their email for a chance to win. A total of 265 comparison group survey responses were retrieved on days when the sheep were not present at the site.

Both groups of respondents were asked to rate their current stress level using a 5-point Likert scale from 1 = very stressed to 5 = not very stressed. A dichotomous variable was created such that “Very stressed” and “Stressed” were coded as “1” and “0” otherwise. Dichotomous demographic variables were also created including Male (1= male and 0 = not male), White (1 = white/Caucasian and 0 = not white/Caucasian), and Undergrad (1 = undergraduate and 0 = otherwise (e.g., graduate student, staff, faculty, community member)).

The group that attended the sheepmower event was also presented with a series of statements and asked to rate them on a 5-point Likert scale from 1 = strongly agree to 5 = strongly disagree. These statements included: (1) Seeing sheep contributed to my happiness, (2) Seeing sheep decreased my feelings of stress, (3) Attending the sheepmower event made me feel less lonely, and (4) Attending the sheepmower event contributed to my well-being. They were also asked the open-ended question, “Is there anything else that you would like us to know?”

#### 2.2.2. Smartphone-Based Data Collection

A second, smartphone-based ecological momentary assessment application was utilized by the research team to collect real-time information about observer self-reported well-being. People watching the sheep were randomly selected by a member of the research team and invited to respond to a single question, “How are you feeling right now?” by selecting one of five “smiley face” emoticons displayed on the researcher’s smartphone, spanning a range of emotions. One week later, when the sheep were not onsite, people who were present at the site were again randomly selected to answer the same question. This intuitive and visual method of using smiley face emojis to represent current levels of well-being encouraged interaction, and the majority of people who were asked to participate agreed.

The research team administered the “smiley face” survey using cell phones on four weekdays in the spring when the sheep were on site (3/31/22, 4/26/22, 4/27/22, and 5/20/22) in the early afternoon between 12 and 2:30 p.m. The research team returned one week later to the same site (4/7/22, 5/3/22, 5/4/22, and 5/27/22) between 12 and 2:30 p.m. to sample people when the sheep were not present. In total, 186 people were randomly asked to select the emojis that matched their feelings on the cell phone survey when the sheep were present and 167 randomly selected people when the sheep were not present. Responses between the two sample groups (sheep and no sheep) were compared to determine differences in overall emotions between the two groups.

### 2.3. Other Data Collection Methods

In addition to participating in the surveys, visitors were encouraged to provide comments about their experience with the sheep through the @UCDavis_sheepmowers Instagram account (which had approximately 2800 followers at the time of data collection). Responses to the prompt to describe their favorite moment with the Sheepmowers were analyzed in this study.

### 2.4. Analyses

Differences in demographic characteristics, life satisfaction, nervousness/anxiety, isolation/loneliness, social connection, ability to concentrate, and current stress level were tested between the sheep grazing observer group (i.e., the Sheepmower group) and comparison group (i.e., the No Sheepmower group) who responded to the online survey using Chi-square tests. Logistic regression analysis was used to examine the association between current stress level and the presence of Sheepmowers while controlling for male/not male, undergraduate status, White/not White.

Responses to the open-ended question on the questionnaire and question prompts collected on the Instagram account were analyzed using thematic analysis. These responses were assigned codes and grouped into themes.

## 3. Results

### 3.1. Characteristics of Web-Based Survey Respondents

There were no significant differences between the two groups that responded to the online surveys with regard to age, race/ethnicity, and time at the university (i.e., number of years affiliated with the university as a student, staff, faculty, or alumnus) (Table 2). About 85% of both groups were between the ages of 18–25 years. Additionally, about half of both had been at the university between two to four years. About a third of both were white/Caucasian and another third were Asian/Pacific Islander.

The two groups were significantly different with respect to gender. The sheepmower group included a greater proportion of people who identified as female (75.1% versus 61.4%). While about two-thirds of both were comprised of undergraduates, there were significant differences between them, with the no sheepmower group consisting of a larger proportion of staff (7.1% versus 3.8%).

### 3.2. Life Satisfaction

In terms of life satisfaction, there was only one significant difference between the two samples (Table 3). It appears that a larger proportion of the no sheepmower group indicated they “never” felt relaxed (8% versus 2%). Almost half of both groups indicated they either “often” or “always” felt satisfied with their lives. Furthermore, almost half of both “often” or “always” felt nervous or anxious. A quarter to a third of the samples felt isolated or had difficulty making friends.

### 3.3. Web-Based and Smart Phone Responses about Feelings of Stress

**Figure 3:**
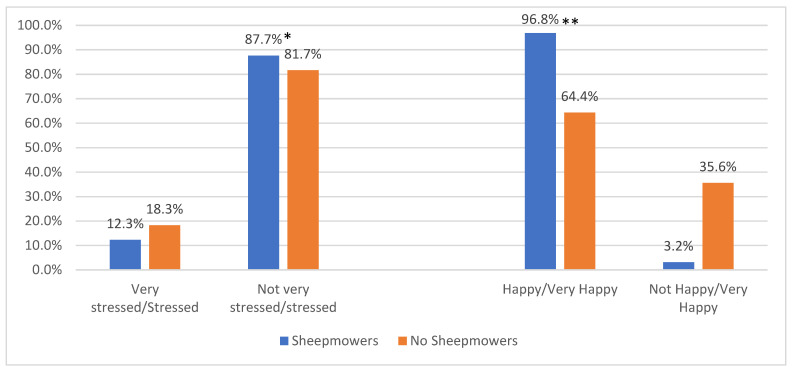
Current Level of Stress at Questionnaire Completion. * Test for differences between Sheepmowers and No Sheepmowers, X^2^(1) = 3.11, *p* = 0.077. ** Test for difference between Sheepmowers and No Sheepmowers, X^2^(1) = 61.72, *p* < 0.001. Note: The level of stress responses were collected from those who completed the web-based survey. The level of happiness responses were collected via smartphone.

There were no significant differences between the two groups with respect to current feelings of being “very stressed” or “stressed” (Figure 1). About 82–87% of both were not “very stressed” or “stressed.” However, there were significant differences between group responses to, “How are you feeling right now?”. The sheepmower group was significantly more likely to endorse a smiley face (96.8%) than the no sheepmower group (64.4%). In contrast, when asked to rate their current level of happiness, there was no significant difference between the groups. About 48.7% of the no sheepmower group indicated they were “very happy/happy” (Figure 3) compared to 54.2% of the sheepmower group.

**Figure 4:**
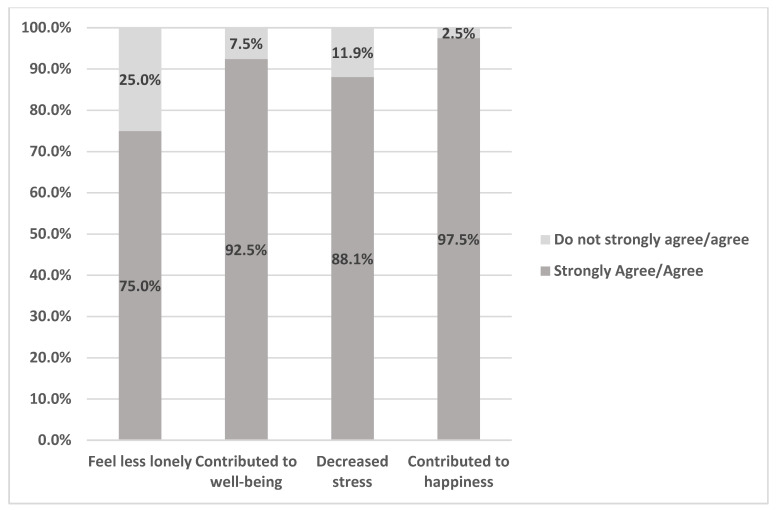
Web-based Survey Respondent Experiences with Sheepmowers.

When asked about their experiences with the sheepmowers, web-based survey respondents indicated that seeing sheep contributed to their happiness (97.5%) and decreased feelings of stress (88.1%) (Figure 4). Additionally, they attributed the event as contributing to their well-being (92.5%) and feeling less lonely (75%).

The logistic regression results (Table 4) indicate there was a significantly lower likelihood of current feelings of being “very stressed” or “stressed’’ among the sheepmower group when compared to the group with no sheepmowers (OR = 0.55, *p* = 0.032). Among males, there was also a significantly lower likelihood of feelings of being “very stressed” or “stressed” (OR = 0.040, *p* = 0.018). The opposite was observed among the undergraduates; there was a significantly higher probability of current feelings of being “very stressed” or “stressed” (OR = 2.49, *p* = 0.031).

### 3.4. What Else the Sheepmower Attendees Wanted to Say

The contribution of sheep to feelings of enhanced mood was reflected in responses to the open-ended question that asked, “Is there anything else you would like us to know?” Respondents expressed their enthusiasm for the sheep and the feelings of respite they brought; remarks included, “I could completely relax”, “I really enjoy sheep as I walk through campus”, and, “I just took a stressful midterm for a class I may or may not pass”. But I told myself I’d see the sheep after, and I’ll feel better.” All the responses to the open-ended question were positive with regard to the sheep.

### 3.5. Feelings of Belonging

There were no significant differences between the sheepmower and no sheepmower groups with respect to their responses to questions about how they perceived the campus. Among the sheepmower group, 72.8% indicated that they felt like they belong on the campus; in contrast, 67.8% of the no sheepmower group reported feeling that way. Additionally, the majority of both groups (sheepmower = 95.5% versus no sheepmower = 91.2%) would describe the campus as being special to them.

### 3.6. Social Media Qualitative Themes

Comments collected from followers of the Sheepmower’s primary social media platform, Instagram, were aggregated into themes that included: (1) Community engagement and education, (2) Place identity, (3) Relaxation effect, and (4) Academic stress reduction.

#### 3.6.1. Community Engagement and Education

One of the themes that emerged suggested that the spectacle of sheep grazing a campus lawn served as a spontaneous catalyst that engaged viewers and created community. As one campus tour guide wrote, “My favorite moment was when I was giving a tour and walked past the lawn; they [the people on the tour] were mesmerized”. Another commenter described seeing everyone’s surprised reaction to the sheep on the first grazing day as her favorite moment. A third shared the impromptu moment with her family: “I sent a photo to my family back home and they were shocked!” The unexpected nature of the event created a shared experience and fostered a sense of community. Commenters described the pleasure they took in watching people engage with the sheep and “seeing students gather around and laugh, smile, and experience peace and joy”. Multiple people wrote about the bonding experience of attending the grazing event with friends, roommates, and even lab mates: “My favorite moment was vibing with the sheep with the rest of the lab”. This level of engagement and sense of community extended even beyond the boundaries of the campus “[The sheepmowers project] has done an incredible job making me care about some sheep eating grass at a school I am not affiliated with!”.

#### 3.6.2. Place Identity

A second theme that arose indicated that one of the effects of the grazing experience, as noted in comments on Instagram, was its contribution to placemaking on campus and enhancement of a communal UC Davis identity. “I was randomly biking to class”, stated one comment, “and saw all the sheep and thought it was the most Davis thing I’ve ever seen!” Another comment referred to the desire to change the campus mascot to a sheep. And, here too, the effect extended beyond the university itself, “even though I’ve never been to Davis, my fave moment has to be finding out the sheepmowers existed. I hope I can come to Davis and visit them someday”.

#### 3.6.3. Relaxation Effect

Despite the fact that no significant differences in stress levels were found between the sheep and no sheep groups among survey respondents, comments posted on the Instagram account indicated that many sheepmower visitors described watching the sheep as a relaxing activity. As one wrote, “My favorite moment is just sitting and watching them all eat and relax together; it helps me relax!”. Another commented, “I loved how cute they were and how they created a place to unwind after class, which is why I tended to stop by them when I had the time”. Simply watching the sheep graze also appeared to have a relaxing effect on viewers “My favorite moment was sitting under an umbrella in one of the lawn chairs and just watching them do their thing”.

#### 3.6.4. Stress Reduction

The final group of responses to the social media prompt asking Instagram followers for their favorite moments with the sheep related to the perceived effects of watching the sheep before or after stressful academic efforts. Some used seeing the sheep to reduce anxiety before exams (“I loved seeing the sheep right before my chem midterm; it helped me distract myself and not stress right before taking the exam”.) and others used the sheep as inspiration to go to class (“My motivation to actually attend my 12p class”.). Multiple people commented on how they loved watching the sheepmowers after “my physics midterm”, “a long day of classes”, and “doing bad on my midterm”. One spoke of the relief the sheep provided “coming out of a midterm and resting under a tree near them”.

## 4. Discussion

Since the 1850s, the Olmstedian model of multifunctionality has been implemented to address growing health and environmental crises through the design of sustainable landscapes that promote human health and well-being and provide respite, beauty, and identity. As knowledge of the healing value of time spent in nature grows, practitioners are increasingly turning towards the concept of biophilic design, a practice that describes modifications to the built environment that satisfy the innate human need to affiliate with nature [47]. Biophilic design focuses on those aspects of nature that, through evolution, have contributed to human health and well-being. Designs that provoke multi-sensory experiences by awakening our senses through smell, touch, sound, and sight enrich everyday life, remind us who we are and where we came from, and lift our spirits, especially when we feel most lost and alone. This best-practices thinking within the discipline of landscape architecture should not remain siloed. As college campuses are reimagining their landscapes to be more inclusive and accessible, there is a great opportunity for healthcare providers to be innovative in how they support the campus community. Similar to Campus Nature Rx programs, Sheepmower programs may help address student mental health through activities that promote connection to the natural world [27].

Our findings corroborate reports of the prevalence of self-reported stress and anxiety in undergraduate college students during the COVID-19 pandemic. They also highlight the potential value of nature-based animal-assisted interventions on campus as a strategy for improving students’ mental health and well-being. High levels of stress, particularly among the undergraduate students sampled in our survey, mirror the findings of other studies examining the mental health of college students. Our results also suggest that high levels of stress may be more prevalent among women, supporting previous research suggesting females are more likely to be negatively affected by the pandemic than their male counterparts [48,49]. Following the analysis of both the web-based survey and smartphone-based smiley face survey, it was observed that while there were no significant differences in the participants’ perceptions of life satisfaction or levels of anxiety, loneliness, and ability to concentrate—in the moment, the presence of sheep provides temporary, noticeable relief and enhanced mood for those who observed the animals.

These findings are supported by the results from the web-based survey questions regarding the direct impact watching the sheep had on the participants. These questions asked participants to reflect on how the presence of sheep influenced their feelings of happiness, stress, and loneliness. The results emphasized that the presence of sheep contributed to increased happiness, decreased feelings of stress, decreased feelings of loneliness, and increased overall well-being.

The informal, outdoor structure of the pilot project may have also contributed to the findings of enhanced moods. Similar to the findings of other studies, the unstructured nature of outreach programs may promote non-threatening environments that facilitate enhanced moods through socialization and participation at a self-guided pace [38]. Likewise, the events were held outdoors, which amid COVID preventative practices, allowed participants to physically distance themselves to a degree that was most comfortable for them, while also being able to socialize and build a sense of community.

The smiley face survey results also suggest the benefit of momentarily improved moods through the presence of sheep. Between the two groups, participants who observed sheep were significantly more likely to endorse a smiley face (96.8%) to reflect their current emotional state; this is in contrast to the absence of sheep (64.4%). It should be noted that these data suggest a burst of temporary respite from negative moods. Mood elevation, however, was not necessarily maintained over the long term, as suggested by the difference in responses to the smiley face data and the survey item embedded within the questionnaire. This disparity might be related to the nature of the smiley face survey having the capacity to capture the spontaneity of the moment, as there was no need to analyze the question being asked. Instead, the respondent was asked to react in the moment.

These data do not allow for conclusions about the long-term effects of sheepmowers on stress or well-being. However, they may contribute to moments of temporary relief. These types of moments are important to allowing people to regroup and consider ways to deal with stress at hand. While characterized as temporary, this project reflects similar results to other research findings surrounding animal-assisted activities. For example, in their meta-analysis, Souter and Miller (2007) [43] concluded that while interventions did not directly cause dramatic improvements to their depression symptoms, patients did report experiencing noticeable psychological relief.

The web-based survey findings suggest that the grazing events did not have a significant effect on participants’ sense of place or overall campus identity. The responses in both survey groups generally reflected similar results, with the majority of participants agreeing/strongly agreeing that they belonged on the UC Davis campus and disagreeing/strongly disagreeing that the UC Davis campus is not special to them. The lack of significant differences may be related to an already strong sense of place among participants. That is, UC Davis is one of the only public universities in the state with strong programs in agriculture and the state’s only Veterinary Medicine school. Thus, the campus has an identity tied to agricultural animals. Nevertheless, the presence of the sheep still seemed to contribute to stress alleviation. Future research exploring the implications of a long-term installation (as opposed to a pilot study) may see alternative findings due to not only the installation being able to reach a larger audience, but also the potential for repeated exposure during that time.

## 5. Limitations

These results should be interpreted in light of limitations within the data and the reliance on pragmatic design methods. For example, the participant sheepmower observers were self-selecting and their experiences may not be generalizable to a broader population. There was a significantly higher number of undergraduates in the sample than any other populations (staff, faculty, community members, graduate students). The fact that survey participants were given an incentive to take the survey (a chance to win a stuffed sheep) could also potentially have influenced the makeup of the groups who responded to the survey and how they responded.

Another limitation was the lack of information about respondents who responded to the “smiley face” question, social media query, and web-based open-ended question. Consequently, the groups who responded to each may not be the same group of people and it is not possible to describe the respondents. Additionally, respondents could have responded to the same questionnaire several times; it was not possible to determine the extent to which this occurred.

Additionally, because the smartphone survey responses were given in the presence of one of the researchers, it is not clear to what extent respondents were influenced in their answers. In future studies, it would be helpful if a station (such as those used at department stores or airports) could be set up to ask a similar question without a researcher present.

A further limitation may be related to the “dose” effect associated with observing the sheepmowers. We do not know exactly how long or how often people attended the event. Additionally, it is possible that there may have been overlap between the respondents who completed surveys on days on which sheep were and were not present. In the future, it would be helpful to add questions to the survey regarding participation in sheepmower events.

This pilot gives evidence that participants found that the sheepmower event contributed to their well-being. The cross-sectional design of the study does not allow for causality to be determined. Future research could build on this study by incorporating standardized scales that could help determine the dimensions of well-being that are affected. Additionally, if cohorts were followed over time, it would be possible to measure pre- and post-exposure well-being levels, control for the amount of exposure to the sheepmowers, and to see how long effects last.

Finally, it may not be possible for all university campuses to have a sheepmower program. However, the concept of utilizing assets a campus already has to deliver both ecological and social benefits to its community can be replicated in other creative ways.

## 6. Conclusions

Traditional academic research on mental health and depression tends to focus on factors related to what humans do to their bodies (drug abuse, sleep deprivation, poor diet and exercise routines, etc.) and less on external environmental factors such as exposure (or lack of exposure) to nature. Research collaborations between public health professionals and environmental designers, however, are increasingly highlighting the role that external environmental factors including wildlife [50], street trees [51], and public parks [52] play in mental health, creating a much-needed dialogue between the fields of ecology, design, and public health. Improvements in personal health and well-being will not come from lectures, social media posts, or scientific articles alone, but from ongoing direct participation. The COVID-19 pandemic posed new mental health challenges on college students—from remote learning to physical distancing—and strategic interventions and programs led by colleges and universities are essential to address these challenges and equip them with applied skills to face new demands on mental health in post-pandemic times. The Sheepmower Project is an intervention example that navigates the implications of COVID-19. The outdoor nature of this project allows for physical distancing while also bringing people together to help build a sense of community. Likewise, this pilot expands on the work of Campus Nature Rx initiatives, which promote spending time in nature and equip college students with skills to reduce emotional distress while simultaneously supporting the environmental health of the campus landscape.

The experience of a land grant university utilizing its own sheep to reduce its environmental impact, and simultaneously promote health and wellness on campus is easy to understand and culturally impactful. It is also physically, visually, and intellectually accessible to college students and the broader public. Partnerships and collaborations such as this help raise awareness about the current crisis in collegiate mental health and promote collaborative, applied solutions drawn from both sustainable environmental design and public health. The success of the Sheepmowers Project relies on creative academic research collaborations between the fields of landscape architecture and public health, input from students and community members, and operational partnerships with planning, design, and management entities. This transdisciplinary effort breaks down traditionally siloed approaches to human and environmental health, focusing instead on a whole-systems approach to developing innovative solutions and encouraging applied collective action. The research supports the belief that a biodiverse environment is also restorative in terms of mental well-being and provides a template for other academic institutions searching for meaningful ways to impact the mental health of its student populations.

## Figures and Tables

**Table 1:** Environmental Conditions on Data Collection Days.

	Sheepmowers		No Sheepmowers	
	Temp (F)	Conditions	Temp (F)	Conditions
Week 1	69	Mostly cloudy	84	Mostly Cloudy
Week 2	74	Fair	77	Fair
Week 3	70	Mostly cloudy	83	Fair
Week 4	79	Fair/windy	79	Mostly cloudy

Source: https://www.wunderground.com (accessed on 3 January 2023).

**Table 2:** Demographic Characteristics of Web-based Survey Respondents.

	Sheepmowers (*n* = 212)		No Sheepmowers (*n* = 262)		Test of Difference between Sheepmowers and No Sheepmowers
	%	*n*	%	*n*	
Gender					
Male	16.92%	34	30.28%	76	X^2^(2) = 11.3214 *p* = 0.003
Female	75.12%	151	61.35%	154	
Non-binary	6.97%	14	6.77%	17	
Age					
<18 years	1.49%	3	1.19%	3	X^2^(5) = 6.7556 *p* = 0.239
18–25	85.07%	171	84.58%	214	
25–34	10.95%	22	9.49%	24	
35–44	0.50%	1	3.56%	9	
45–54	0.50%	1	0.79%	2	
>55 years	1.49%	3	0.40%	1	
Race/Ethnicity *					
Asian or Pacific Islander	40.79%	93	33.88%	103	X^2^(5) = 4.6695 *p* = 0.458
Black or African American	1.75%	4	1.32%	4	
Hispanic, Latino, or Spanish	15.79%	36	18.75%	57	
Native American, American Indian, or Alaska Native	1.75%	4	0.66%	2	
White or Caucasian	35.09%	80	37.50%	114	
Other	3.51%	8	4.93%	15	
Affiliation with University *					
Undergraduate	73.11%	155	75.19%	200	X^2^(6) = 13.2191 *p* = 0.040
Graduate	12.74%	27	10.15%	27	
Staff	3.77%	8	7.14%	19	
Faculty	0.47%	1	2.26%	6	
Local Community Member	2.83%	6	0.38%	1	
Visitor	3.77%	8	3.76%	10	
Alumni	3.30%	7	1.13%	3	
Length of Time at University					
<1 year	21.39%	43	21.51%	54	X^2^ (4) = 0.4039 *p* = 0.354
About 1 year	16.42%	33	23.51%	59	
2–4 years	52.24%	105	47.81%	120	
5–7 years	5.97%	12	4.78%	12	
>7 years	3.98%	8	2.39%	6	

Note: * respondents asked to choose all responses that applied.

**Table 3:** Web-based Survey Respondents’ Overall Life Satisfaction.

	Sheepmowers (S)										No Sheepmowers (NS)										Test of Difference between S and NS
	Never		Rarely		Sometimes		Often		Always		Never		Rarely		Sometimes		Often		Always		
	%	*n*	%	*n*	%	*n*	%	*n*	%	*n*	%	*n*	%	*n*	%	*n*	%	*n*	%	*n*	
I feel satisfied with my life.	0.49%	1	7.39%	15	42.86%	87	41.38%	84	7.88%	16	1.51%	4	4.91%	13	42.64%	113	41.89%	111	9.06%	24	X^2^(4) = 2.49 *p* = 0.646
I feel nervous or anxious.	0.99%	2	3.45%	7	35.47%	72	45.32%	92	14.78%	30	1.15%	3	8.40%	22	32.82%	86	44.27%	116	13.36%	35	X^2^(4) = 4.95 *p* = 0.293
I feel alone or isolated from other people.	4.93%	10	19.70%	40	46.31%	94	25.12%	51	3.94%	8	2.66%	7	24.33%	64	46.77%	123	20.91%	55	5.32%	14	X^2^ (4) = 4.11 *p* = 0.391
It is difficult for me to make friends or connect with other people.	8.87%	18	26.11%	53	44.33%	90	16.75%	34	3.94%	8	6.46%	17	29.66%	78	42.21%	111	17.11%	45	4.56%	12	X^2^ (4) = 1.63 *p* = 0.804
I can concentrate on tasks that I need to.	1.97%	4	10.34%	21	45.81%	93	35.96%	73	5.91%	12	3.80%	10	14.83%	39	41.44%	109	34.60%	91	5.32%	14	X^2^ (4) = 3.70 *p* = 0.448
I feel relaxed.	1.97%	4	26.60%	54	46.80%	95	22.17%	45	2.46%	5	8.02%	21	23.66%	62	45.80%	120	18.70%	49	3.82%	10	X^2^ (4) = 9.52 *p* = 0.049

**Table 4:** Logistic Regression Results for Outcome: Currently Very Stressed/Stressed.

	Odds Ratio	95% Confidence Interval	*p*-Value
Sheepmower	0.55	0.31, 0.95	0.032
Undergraduate	2.49	1.085, 5.72	0.031
Male	0.40	0.19, 0.85	0.018
White	1.39	0.79, 2.43	0.25
Constant	0.12	0.048, 0.27	<0.001

Note: Pseudo R2 = 0.0474, *n* = 443.

## Data Availability

Not applicable.

## References

[1] Birney Vickery M. (2021). Landscape and Infrastructure: Reimagining the Pastoral Paradigm for the Twenty-First Century. Crit. D’art Livres Par Auteur..

[2] Handler R., Pearce J.M. (2022). Greener sheep: Life cycle analysis of integrated sheep agrivoltaic systems. Clean. Energy Syst..

[3] Yoshihara Y., Miyagawa Y., Sakai M. (2021). Challenging sheep grazing in orchards: Changes in nutrition, performance, and the health of animals and the effects on the vegetation and soil. Grassl. Sci..

[4] Niles M.T., Garrett R.D., Walsh D. (2018). Ecological and economic benefits of integrating sheep into viticulture production. Agron. Sustain. Dev..

[5] Colley T.A., Olsen S.I., Birkved M., Hauschild M.Z. (2019). Delta Life Cycle Assessment of Regenerative Agriculture in a Sheep Farming System. Integr. Environ. Assess. Manag..

[6] Colantoni A., Egidi G., Quaranta G., D’Alessandro R., Vinci S., Turco R., Salvati L. (2020). Sustainable Land Management, Wildfire Risk and the Role of Grazing in Mediterranean Urban-Rural Interfaces: A Regional Approach from Greece. Land.

[7] Lenaghan M.A. (2016). Sheep grazing in ‘lawnscape’ management: An emissions comparison with conventional ‘lawnscape’management. Landsc. Res..

[8] Liu C.H., Stevens C., Wong S.H., Yasui M., Chen J.A. (2018). The prevalence and predictors of mental health diagnoses and suicide among U.S. college students: Implications for addressing disparities in service use. Depression Anxiety.

[9] Pedrelli P., Nyer M., Yeung A., Zulauf C., Wilens T. (2014). College Students: Mental Health Problems and Treatment Considerations. Acad. Psychiatry.

[10] Batra K., Sharma M., Batra R., Singh T., Schvaneveldt N. (2021). Assessing the Psychological Impact of COVID-19 among College Students: An Evidence of 15 Countries. Healthcare.

[11] O’Connor R.C., Wetherall K., Cleare S., McClelland H., Melson A.J., Niedzwiedz C.L., O’Carroll R.E., O’Connor D.B., Platt S., Scowcroft E. (2021). Mental health and well-being during the COVID-19 pandemic: Longitudinal analyses of adults in the UK COVID-19 Mental Health & Wellbeing study. Br. J. Psychiatry.

[12] Oswalt S.B., Lederer A.M., Chestnut-Steich K., Day C., Halbritter A., Ortiz D. (2020). Trends in college students’ mental health diagnoses and utilization of services, 2009–2015. J. Am. Coll. Health.

[13] Elmer T., Mepham K., Stadtfeld C. (2020). Students under lockdown: Comparisons of students’ social networks and mental health before and during the COVID-19 crisis in Switzerland. PLoS ONE.

[14] Holm-Hadulla R.M., Koutsoukou-Argyraki A. (2015). Mental health of students in a globalized world: Prevalence of complaints and disorders, methods and effectivity of counseling, structure of mental health services for students. Ment. Health Prev..

[15] Bara Stolzenberg E., Aragon M.C., Romo E., Couch V., McLennan D., Eagan M.K., Kang N. (2020). The American Freshman: National Norms Fall 2019.

[16] Khan S., Siddique R., Li H., Ali A., Shereen M.A., Bashir N., Xue M. (2020). Impact of coronavirus outbreak on psychological health. J. Glob. Health.

[17] Browning M.H.E.M., Larson L.R., Sharaievska I., Rigolon A., McAnirlin O., Mullenbach L., Cloutier S., Vu T.M., Thomsen J., Reigner N. (2021). Psychological impacts from COVID-19 among university students: Risk factors across seven states in the United States. PLoS ONE.

[18] Chirikov I., Soria K.M., Horgos B., Jones-White D. (2020). Undergraduate and graduate students’ mental health during the COVID-19 pandemic. SERU Consort..

[19] Kaparounaki C.K., Patsali M.E., Mousa D.-P.V., Papadopoulou E.V., Papadopoulou K.K., Fountoulakis K.N. (2020). University students’ mental health amidst the COVID-19 quarantine in Greece. Psychiatry Res..

[20] McAlpine K.J. (2021). Depression, Anxiety, Loneliness are Peaking in College Students. Brink.

[21] Oh H., Marinovich C., Rajkumar R., Besecker M., Zhou S., Jacob L., Koyanagi A., Smith L. (2021). COVID-19 dimensions are related to depression and anxiety among US college students: Findings from the Healthy Minds Survey 2020. J. Affect. Disord..

[22] Zhai Y., Du X. (2020). Addressing collegiate mental health amid COVID-19 pandemic. Psychiatry Res..

[23] Wang Z.H., Yang H.L., Yang Y.Q., Liu D., Li Z.-H., Zhang X.-R., Zhang Y.-J., Shen D., Chen P.-L., Song W.-Q. (2020). Prevalence of anxiety and depression symptom, and the demands for psychological knowledge and interventions in college students during COVID-19 epidemic: A large cross-sectional study. J. Affect. Disord..

[24] Cohen K.A., Graham A.K., Lattie E.G. (2020). Aligning students and counseling centers on student mental health needs and treatment resources. J. Am. Coll. Health.

[25] Sanchez D., King-Toler E. (2007). Addressing disparities: Consultation and outreach strategies for university settings. Consult. Psychol. J. Pract. Res..

[26] Marks L.I., McLaughlin R.H. (2005). Outreach by College Counselors: Increasing Student Attendance at Presentations. J. Coll. Couns..

[27] Rakow D.A., Ibes D.C. (2022). Campus Nature Rx: How investing in nature interventions benefits college students. Front. Psychol..

[28] Ulrich R.S. (1979). Visual landscapes and psychological well-being. Landsc. Res..

[29] Bratman G.N., Daily G.C., Levy B.J., Gross J.J. (2015). The benefits of nature experience: Improved affect and cognition. Landsc. Urban Plan..

[30] Thompson C.W., Aspinall P., Roe J., Robertson L., Miller D. (2016). Mitigating Stress and Supporting Health in Deprived Urban Communities: The Importance of Green Space and the Social Environment. Int. J. Environ. Res. Public Health.

[31] Frumkin H., Bratman G.N., Breslow S.J., Cochran B., Kahn P.H., Lawler J.J., Levin P.S., Tandon P.S., Varanasi U., Wolf K.L. (2017). Nature contact and human health: A research agenda. Environ. Health Perspect..

[32] James J.J., Christiana R.W., Battista R.A. (2019). A historical and critical analysis of park prescriptions. J. Leis. Res..

[33] Ibes D.C., Forestell C.A. (2020). The role of campus greenspace and meditation on college students’ mood disturbance. J. Am. Coll. Health.

[34] Ibes D., Hirama I., Schuyler C. (2018). Greenspace Ecotherapy Interventions: The Stress-Reduction Potential of Green Micro-Breaks Integrating Nature Connection and Mind-Body Skills. Ecopsychology.

[35] Kiers A.H., Rakow D.A., Parker S., Dewa C.S. (2021). A pilot study on the potential for formalized nature-based instruction to mitigate stress and increase social bonds in university students. J. Am. Coll. Health.

[36] Jackson S.B., Stevenson K.T., Larson L.R., Peterson M.N., Seekamp E. (2021). Outdoor activity participation improves adolescents’ mental health and well-being during the COVID-19 pandemic. Int. J. Environ. Res. Publ. Health.

[37] Javelle F., Laborde S., Hosang T.J., Metcalfe A.J., Zimmer P. (2021). The importance of nature exposure and physical activity for psychological health and stress perception: Evidence from the first lockdown period during the Coronavirus Pandemic 2020 in France and Germany. Front. Psychol..

[38] Stewart L.A., Dispenza F., Parker L., Chang C.Y., Cunnien T. (2014). A Pilot Study Assessing the Effectiveness of an Animal-Assisted Outreach Program. J. Creat. Ment. Health.

[39] Daltry R., Mehr K. (2015). Therapy Dogs on Campus: Recommendations for Counseling Center Outreach. J. Coll. Stud. Psychother..

[40] Wood E., Ohlsen S., Thompson J., Hulin J., Knowles L. (2017). The feasibility of brief dog-assisted therapy on university students stress levels: The PAwS study. J. Ment. Health.

[41] Berget B., Ekeberg Ø., Braadstad B.O. (2008). Attitudes to animal-assisted therapy with farm animals among health staff and farmers. J. Psychiatr. Ment. Health Nurs..

[42] Berget B., Ekeberg Ø., Pedersen I., Braastad B.O. (2011). Animal-Assisted Therapy with Farm Animals for Persons with Psychiatric Disorders: Effects on Anxiety and Depression, a Randomized Controlled Trial. Occup. Ther. Ment. Health.

[43] Souter M., Miller M. (2007). Do Animal-Assisted Activities Effectively Treat Depression? A Meta-Analysis. Anthrozoös.

[44] Sackett D.L. (2013). Clinicial-Trialist Rounds: 16. Mind Your Explanatory and Pragmatic Attitudes-Part 1: What?. Clin. Trials..

[45] Sackett D.L. (2013). Clinicial-Trialist Rounds: 17. Mind Your Explanatory and Pragmatic Attitudes-Part 1: How?. Clin. Trials..

[46] Zudgeist M.G.P., Goetz I., Groenwold R.H., Irvin E., van Thiel G.J., Grobbee D.E., Package G.W. (2017). Series: Pragmatic Trials and Real World Evidence: Paper 1. Introduction. J. Clin. Epidemiol..

[47] Kellert S.R., Wilson E.O. (2008). Biophilia. Hum. Ecol..

[48] Liu N., Zhang F., Wei C., Jia Y., Shang Z., Sun L., Wu L., Sun Z., Zhou Y., Wang Y. (2020). Prevalence and predictors of PTSS during COVID-19 outbreak in China hardest-hit areas: Gender differences matter. Psychiatry Res..

[49] Wenham C., Smith J., Morgan R. (2020). Gender and COVID-19 Working Group. COVID-19: The gendered impacts of the outbreak. Lancet.

[50] Hammoud R., Tognin S., Burgess L., Bergou N., Smythe M., Gibbons J., Davidson N., Afifi A., Bakolis I., Mechelli A. (2022). Smartphone-based ecological momentary assessment reveals mental health benefits of birdlife. Sci. Rep..

[51] Wolf K.L., Lam S.T., McKeen J.K., Richardson G.R., van den Bosch M., Bardekjian A.C. (2020). Urban trees and human health: A scoping review. Int. J. Environ. Res. Public Health.

[52] Wood L., Hooper P., Foster S., Bull F. (2017). Public green spaces and positive mental health–investigating the relationship between access, quantity and types of parks and mental wellbeing. Health Place.

